# Role of thermal physiology and bioenergetics on adaptation in tree shrew (*Tupaia belangeri*): the experiment test

**DOI:** 10.1038/srep41352

**Published:** 2017-02-01

**Authors:** Lin Zhang, Fang Yang, Zheng-kun Wang, Wan-long Zhu

**Affiliations:** 1 Key Laboratory of Ecological Adaptive Evolution and Conservation on Animals-Plants in Southwest Mountain Ecosystem of Yunnan Province Higher Institutes College, School of Life Sciences, Yunnan Normal University, Kunming 650500, China; 2 School of Laboratory Medicine, Hubei University of Chinese Medicine, Wuhan 430065, China

## Abstract

Ambient conditions, as temperature and photoperiod, play a key role in animals’ physiology and behaviors. To test the hypothesis that the maximum thermal physiological and bioenergetics tolerances are induced by extreme environments in *Tupaia belangeri*. We integrated the acclimatized and acclimated data in several physiological, hormonal, and biochemical markers of thermogenic capacity and bioenergetics in *T. belangeri*. Results showed that *T. belangeri* increased body mass, thermogenesis capacity, protein contents and cytochrome *c* oxidase (COX) activity of liver and brown adipose tissue in winter-like environments, which indicated that temperature was the primary signal for *T. belangeri* to regulate several physiological capacities. The associated photoperiod signal also elevated the physiological capacities. The regulations of critical physiological traits play a primary role in meeting the survival challenges of winter-like condition in *T. belangeri*. Together, to cope with cold, leptin may play a potential role in thermogenesis and body mass regulation, as this hormonal signal is associated with other hormones. The strategies of thermal physiology and bioenergetics differs between typical Palearctic species and the local species. However, the maximum thermal physiology and bioenergetic tolerance maybe is an important strategy to cope with winter-like condition of *T. belangeri*.

Phenotypic plasticity is the ability of individuals to change phenotype with fluctuations in climate conditions, and responses mainly include aspects of morphology, physiology, behavior, and phenology^1^. The energy metabolism of small mammals may be the most suitable field for studying phenotypic plasticity changes^2^. The variation in physiological traits in individuals is an important response to the environment^1^,^3^,^4^. Climate conditions, including temperature and photoperiod, play a key role in seasonal variation in body mass, food intake, body fat mass, and other traits^5^,^6^,^7^,^8^,^9^ for many species inhabiting in temperature zone, especially mammals. However, different species show different responses to climate conditions, such as *Phodopus sungorus*^6^, *Ochotona curzoniae*^10^, *Microtus oeconomus*^10^, and *Clethrionomys glareolus*^11^.

Small mammals’ response to seasonal variations such as cold or short photoperiods, by regulating physiological strategies, such as body mass and thermogenic capacity (e.g., nonshivering thermogenesis, NST)^6^,^7^,^8^,^12^,^13^,^14^,^15^,^16^,^17^,^18^,^19^,^20^,^21^,^22^,^23^. The brown adipose tissue (BAT) is a specialized organ that is involved in NST^24^,^25^,^26^, and in increased energy expenditure involves the hypothalamic-pituitary-thyroid axis and sympathetic nervous system^27^. The NST capacity entirely depends on uncoupling protein 1(UCP1) in BAT, a 32-kD carrier protein located in the inner membrane of mitochondria, that separates oxidative phosphorylation from adenosine triphosphate synthesis, with energy dissipated as heat^27^. Most previous researches focused on the influence of temperature/photoperiod on physiological and biochemical properties of individuals, such as *Sekeetamys calurus*^20^, *Lasiopodomys brandtii*^21^, *Meriones unguiculatus*^21^, *Eothenomys miletus*^22^, *Dromiciops gliroides*^28^, *Apodemus sylvaticus*^29^, *Acomys cahirinus*^30^,^31^, *Dipodomys ordii*^32^, *Dicrostonyx groenlandicus*^33^, *Mesocricetus auratusz*^34^, *Microtus agrestis*^35^, and *Phodopus sungorus*^36^. The cytochrome *c* oxidase (COX, complex IV) represents the terminal enzyme of oxidative phosphorylation in mitochondria and is involved in mitochondrial energy metabolism^37^, and other biochemical acitivities associated with metabolism. In small mammals, hyperplasia expends energy expenditure with an increase of thermogenesis during winter-like condition^38^. Within the arcuate nucleus, there are two types of neuropeptides: (1). anorectic neuropeptides (pro-opiomelanocortin: POMC, and cocaine- and amphetamine-regulated tran-script: CART), and (2). orexigenic neuropeptides (neuropeptide Y: NPY, and agouti-related protein: AgRP). The balance between these four neuropeptides can inhibit food intake and stimulate energy expenditure: by stimulating NPY/AgRp-expression and suppression POMC/CART-expression, the food intake increased and energy expenditure decreased^39^, such as *Apodemus chevrieri*^40^ and *Eothenomys oliter*^23^, and the mRNA levels appear in seasonal cycles, such as *E. oliter*, the NPY/AgRp mRNA level appear the peak and bottom at winter and summer, respectively, however, the POMC/CART mRNA level appear an opposite trand^23^.

Tree shrew, *Tupaia belangeri* (Mammalia: Scandentia: Tupaiidae), a squirrel-liked lower primate, is a unique Oriental species, and is increasingly being used as a new and promising animal model in biomedical research^41^. This animal originates from the tropical island and is a widespread across Southeast-Asia and north of the isthmus of Kra, including Southern China, and the Yunnan-Kweichow Plateau is the northern limit of its distribution^42^. Specifically, the cold environment reduces serum leptin levels but enhances thermogenesis capacity in this species^19^,^43^,^44^, whereas the short photoperiod enhances thermogenesis capacity^45^. Here, we hypothesize that the extreme condition induces the maximum physiological capacity and affect the strategies of resistance and avoidance in *T. belangeri*, and this capacity plays a key role in regulation of body mass and thermoregulation in *T. belangeri*.

The present study was designed to investigate the physiological traits, hormones level, genes expression and biochemical processes in captive seasonally acclimatized and temperature/photoperiod acclimated *T. belangeri* to test us hypothesizes. This work wants to get results in: (1) to establish how *T. belangeri* physiological strategies for physiological capacity regulation were influenced during the different environment? (2) to examine how physiological traits and biochemical processes are associated in *T. belangeri*? and (3) to investigate how fluctuations in climate conditions affect hormone levels and gene (NPY, POMC and CART) expression in *T. belangeri*? We predicted that *T. belangeri* would increase in body mass and increase the biochemical activity associated with thermogenesis capacity during the cold and/or short photoperiod conditions, and that thermogenesis capacity would plateau in response to winter-like conditions. However, in summer-like conditions, we expected that the thermogenesis capacity and body mass would decrease, and food intake would be limited. Moreover, leptin may play a role on body mass regulation by acting on NPY/AgRp and POMC/CART mRNA expression in *T. belangeri*.

## Materials and Methods

### Ethics Statement

This research was performed in accordance with the NIH *Guide for the Principles of Animal Care* and Law of the Protection of wildlife in China. The protocol and study were approved by the Animal Care and Use Committee of the School of Life Sciences, Yunnan Normal University (No.: 13-0901-011).

### Animals and culture

The tree shrews, *T. belangeri*, were captured (25°25′–26°22′ N, 102°13′–102°57′ E, at 1679 m altitude) at the boscage of Luquan County, Yunnan Province, China, and maintained at the School of Life Sciences, Yunnan Normal University, Kunming (1910 m altitude). All animals were healthy adults, and housed individually in a wire cage (40 cm × 40 cm × 40 cm), water with vitamin were provided *ad libitum*. The cage environment was maintained at 12 L: 12D (lights on at 08:00), 25 ± 1 °C, and 65–92% relative humidity. The shrews were fed a food mixture containing 25.0% crude protein, 6.3% crude fat, 4.6% crude fiberd, 7.4% ash, and 0.96 KJ/g gross energy, as well as apples, pears and other fruits twice weekly^36^.

### Experiment design

#### Experiment 1: seasonal acclimatization

96 tree shrews were wild-captured in mid-July and October 2011, mid-January and late April 2012 (hereafter referred to the summer, autumn, winter and spring groups, respectively). The animals were stabilized for more than 2 weeks before testing for seasonal variation in body mass, food intake, and thermogenesis. A total of 35 adult tree shrews, except pregnant or lactating individuals, were used in the present study (summer, n = 10; autumn, n = 9; winter, n = 8; spring, n = 8, each group included 4–5 females and 4–5 males). Between capture and metabolic analysis, the animals were kept individually in a metabolic cage (40 × 40 × 40 cm^3^) in a room with natural temperature and photoperiod.

#### Experiment 2: temperature and photoperiod acclimation

The experiment was conducted in January 2011. In order to test singular and/or associated effects of ambient temperature and photoperiod on body mass, food intake, and thermogenesis, 40 adult weight-matched tree shrews were housed individually (maintained at 12 L: 12D (lights on at 08:00am), 25 ± 1 °C, and 85–92% relative humidity), and kept for at least 2 weeks before experiment. After the acclimatizing period, the animals were randomly assigned to the following four groups: long photoperiod (LD, 16 L: 8D) with cold (5 ± 1 °C), LD with warm (30 ± 1 °C), short photoperiod (SD, 8 L: 16D) with cold, SD with warm; each group included 10 individuals (5 females and 5 males). Animals were acclimated for four weeks. Body mass, food intake, and metabolic rate were monitored weekly.

### Mitochondria respiration

Following the measurements of metabolic traits^46^, animals were killed between 12:00 and 14:00 by decapitation and blood was collected for hormone determination. The blood was centrifuged at 4,000 rpm for 30 min after a 30-min interval and the serum was collected and stored at −72 °C for hormone determination. Liver, BAT and testicular tissues were carefully and quickly removed and weighted (0.1 mg), and their adhering tissues separated. The organs were blotted, weighed, and placed in ice-cold sucrose-buffered medium and then homogenized to isolate mitochondria^47^. The protein content of mitochondria was determined using the Folin phenol method with bovine serum albumin as standard^48^. The state 4 (ST_4_) of mitochondrial respiration of liver and BAT were measured by Hanstech Oxy-Lab Chloroab 2 oxygen electrode (Hansatech Instruments LTD., England).

### Enzyme activity

The COX (EC 1.9.3.1) activity was measured using the polarographic method using an oxygen electrode (Hansatech Instruments Ltd., England)^49^,^50^, Thyroxin 5′-deiodinase (T_4_ 5′-DII; EC 1.97.1.10) activity in BAT was assayed as previously described^51^.

### Uncoupling protein 1 (UCP1)

UCP1 content was measured using Western blotting as described previously^8^,^50^. Total BAT protein (15 μg per lane) was separated in a discontinuous SDS-polyacrylamide gel (12.5% running gel and 3% stacking gel) and blotted to a nitrocellulose membrane (Hybond-C, Amersham Biosciences, England). The gels and nitrocellulose membranes were stained with Coomassie brilliant blue and Ponceau S (red), respectively to test the efficiency of protein transfer. Unspecific binding sites were saturated with 5% nonfat dry milk in PBS. UCP1 was detected using a polyclonal rabbit anti-hamster UCP1 (1:5000) as a primary antibody and peroxidase-conjugated goat anti-rabbit IgG (1:5000) (Jackson Immuno. Inc., USA) as the second antibody. Enhanced chemoluminescence (ECL, Amersham Biosciences, England) was used for detection. UCP1 concentration was determined from area readings using Scion Image Software (Scion Corporation) and was expressed as relative units (RU)^8^,^50^.

### Hormone concentration

Serum leptin levels were determined by radioimmunoassay (RIA) with the ^125^I Multi-Species Kit (St. Louis), and their values were obtained in a single RIA. The lowest level of leptin detected by this assay was 1.0 ng/ml when using a 100-μl sample size (instructions for Multi-Species Kit). The inter- and intra-assay variabilities for leptin RIA were <3.6% and 8.7%, respectively.

The concentrations of triiodothyronine (T_3_), thyroxine (T_4_), thyroid-stimulating hormone (TSH), and testosterone (T) in the serum were determined using RIA kits (China Institute of Atomic Energy). These kits were validated for all species tested by cross-activity. Intra- and inter-assay coefficients of variation were 2.4% and 8.8% for the T_3_, 4.3% and 7.6% for T_4_, 3.6% and 6.9% for TSH, and 7.6% and 8.1% for T, respectively.

### Measurement of body fat mass

After dissection of the hypothalamus and BAT, internal organs were removed and the eviscerated carcass was weighed and oven dried at 60 °C to constant weight, the dry carcass mass (W_1_) was then weighed. After grinding the dry carcass in a mill and mixing it completely, 1 g of sample (W_2_) was weighed to an accuracy of ±1 mg into a thimble (W_3_). Body fat extraction was performed in a Soxtec^TM^ 2043 Fat Extraction Systems (FOSS, Hilleroed, Denmark) with petroleum ether. Subsequently, the thimble containing the residual sample was oven dried at 60 °C to constant weight and weighed (W_4_). Finally, the carcass fat mass was calculated using the formula:


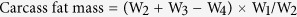


### Real-time reverse transcription-polymerase chain reaction (RT-PCR) assay of hypothalamic gene expression of NPY, POMC and CART

#### Primer design

Real-time qRT-PCR was used to assay hypothalamic gene expression of NPY, POMC, and CART. Species-specific primer sets for NPY, POMC, CART, and Beta-actin in tree shrews were designed according to the gene sequences of rats and mice stored in Genbank (Table 1).

#### Total RNA isolation and cDNA synthesis

Total RNA was isolated from the hypothalamus using the TRIzol Kit (Invitrogen, Carlsbad, CA, USA) according to the manufacturer protocols. To remove any contaminants, DNA and RNA samples were treated with DNase I (Promega, USA) for 30 min at 37 °C, followed by another cycle of TRIzol extraction to eliminate residual DNase I. An equal amount (3 lg) of total RNA for each sample was transcribed into first strand cDNA using M-MLV First Strand Kit (Invitrogen, Carlsbad, CA, USA) according to the manufacturer instructions.

#### Real-time qPCR

Primers set for bata-actin and three hypothalamic genes were designed for real-time PCR (Table 1). Real-time PCR was completed using the SYBR Green I qPCR kit (TaKaRa Bio, Dalian, China) in the Mx3000 P quantitative PCR system (Stratagene, La Jolla, CA, USA). Real-time RT-PCR was carried out in 12.5 μL reaction agent comprised of 6.25 μL 2 SYBR Premix EX Taq master mix, 1 μL cDNA and 0.25 μL of each primer (10 umol/L). Thermal cycling conditions were: 95 °C for 20 s, 35 cycles of 95 °C for 20 s, 56 °C for 50 s, and 72 °C for 90 s, then 72 °C for 10 min. Melting curve analysis showed a single PCR product after amplification of three hypothalamic genes and bata-actin, and end products of PCR were further confirmed by DNA sequencing. We constructed standard curves for each gene via serial dilutions of cDNA (2-fold dilutions). Analysis of standard curves between target genes and bata-actin showed that they had similar amplification efficiency, which ensures the validity of comparative quantity method. The data derived from Mx3000 P quantitative software were expressed as relative amounts. Gene expression was calculated by the 2^−ΔΔCt^ method^52^.

#### Statistical analysis

Data were analyzed using SPSS 16.0 software (SPSS Inc., Chicago, IL, USA). Since no sexual effects were found on almost all measured parameters, data from females and males were combined. Prior to all statistical analyses, data were tested for assumptions of normality and homogeneity of variance using the Kolmogorov–Smirnov and Levene tests, respectively. Seasonal variation such as COX activity, mitochondrial protein content, thyroid hormones and UCP1 were analyzed using one-way analysis of variance (ANOVA). Two-way ANCOVA was used to detect the effect of photoperiod and temperature on thermogenic properties in liver, BAT and hormones, using body mass as the covariate. Differences among groups were detected using Duncan’s Multiple Range test. Results are presented as mean ± SME (n sample size) in the test, and *P* < 0.05 was considered as statistically significant.

## Results

### Experiment 1: seasonal acclimatization

#### Changes in mitochondrial protein content, COX activity and UCP1 content

There was a significant difference in the absolute mass of BAT (Table 2), the mitochondrial protein content (M_t_P), COX activity and UCP1 in BAT varied significantly across seasons (Table 2), the M_t_P and COX activity were markedly higher in winter than in other seasons, the COX activity was 159.64% in winter higher than in summer, UCP1 was 68% in winter higher than in summer.

There was a significant difference in the absolute mass of Liver (Table 2), the M_t_P and COX activity in liver varied significantly across seasons (Table 2), the M_t_P and COX activity were markedly in winter higher than in the other seasons, the COX activity was 11.93% in winter higher than in summer.

#### Changes in serum hormones, body fat mass and hypothalamic expression of genes regulating appetite

**Figure 1:**
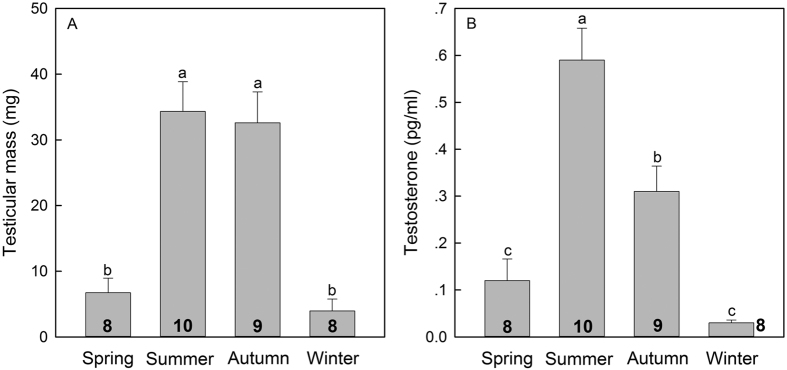
Seasonal variations of testicular mass (**A**) and testosterone (**B**) of tree shrews. Results are presented as mean ± SME. Means with different letters differ significantly (Tukey’s post hoc test, α = 0.05; a > b > c). Numbers inside bars indicate sample size for each season.

**Figure 2:**
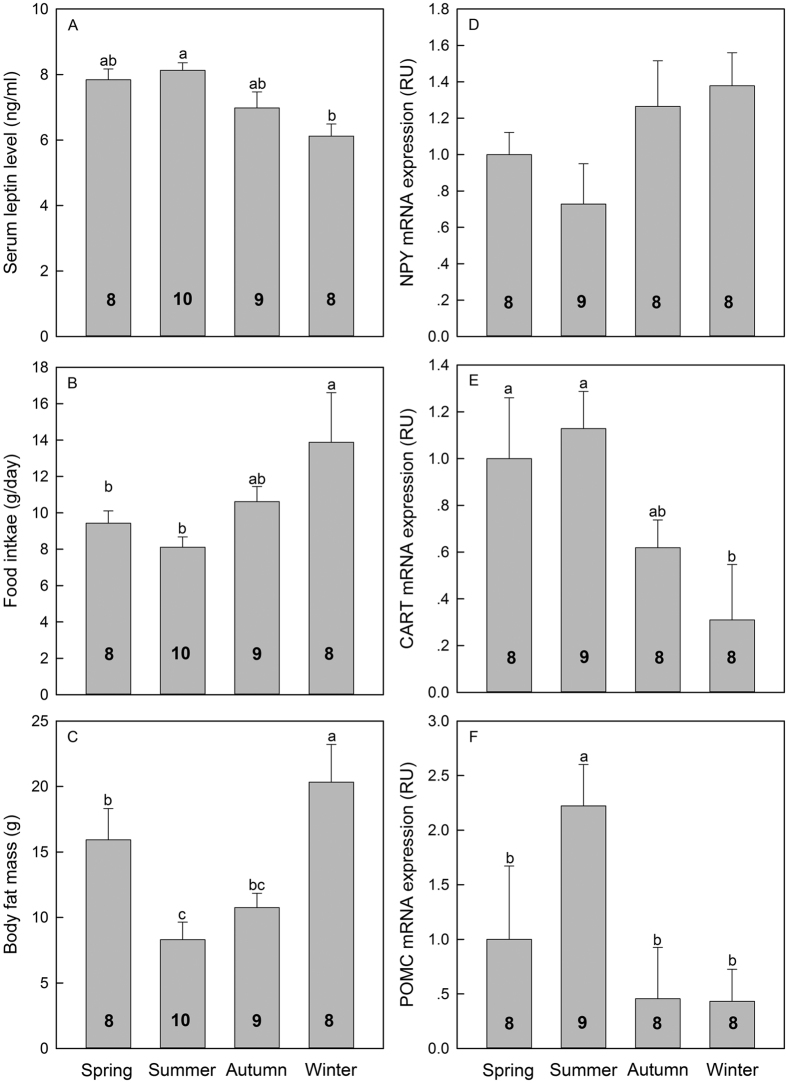
Seasonal variations of serum leptin levels (**A**), food intake (**B**), body fat mass (**C**) and hypothalamic gene ((**D**) NPY, neuropeptide Y; (**E**) CART; cocaine- and amphetamine-regulated transcript; (**F**) POMC, pro-opio-melanocortin) expression of tree shrews. Results are presented as mean ± SME. Means with different letters differ significantly (Tukey’s post hoc test, α = 0.05; a > b > c). Numbers inside bars indicate sample size for each season.

Seasonal variations were also detected in testicular mass (*F* = 4.21, *P* < 0.05, Fig. 1A), which was significantly lower in winter than in summer. Seasonal variation was also detected inT_3_ (Table 2) and T levels (*F* = 3.86, *P* < 0.05, Fig. 1B). For example, T_3_ level was markedly lower in summer (0.74 ± 0.04 ng/ml) than in winter (1.38 ± 0.19 ng/ml). Serum T_4_, and TSH levels gradually decreased from warmer to colder months. The serum leptin was significantly in summer (8.12 ± 0.23 ng/ml) higher than in winter (6.11 ± 0.37 ng/ml) (*P* < 0.05, Fig. 2A), however, the body fat mass was markedly lower in summer (8.3 ± 1.3 g) than in winter (20.3 ± 2.9 g) (*P* < 0.05, Fig. 2B). Therefore, there was an opposite seasonal trend of serum leptin and body fat mass in tree shrews (Fig. 2A,B,C). In all four seasons, a significantly higher value of mRNA expression of hypothalamic POMC (*F* = 7.433, *P* < 0.05; Fig. 2F) and CART (*F* = 3.856, *P* < 0.05; Fig. 2E) was observed, while no significant changes were observed in the mRNA expression of hypothalamic NPY (*F* = 2.147, *P* > 0.05; Fig. 2D) across seasons.

### Experiment 2: temperature and photoperiod acclimation

#### Effect of photoperiod and/or temperature on mitochondrial protein content and COX activity

A significant interaction between photoperiod and temperature was observed for the body mass, total protein, mitochondrial protein, ST_4_, and COX activity in liver (Table 3). The BAT mass was influenced only by temperature (Table 3). However, the total protein, mitochondrial protein, ST_4_, COX activity, T_4_ 5′-DII activity, and UCP 1 in BAT were affected by both photoperiod and temperature, or by the interaction between the two (Table 3).

#### Effect of photoperiod and/or temperature on serum hormones

Both temperature and photoperiod, and their interaction, had significant effects on T_3_ and TSH levels in tree shrews (Table 3); however, there were no effects of photoperiod or the interaction of photoperiod and temperature on serum leptin level or T_4_ levels (Table 3).

## Discussion

Ambient conditions, such as temperature and photoperiod, play a key role in animals’ physiology and behaviors^44^. Our results showed that *T. belangeri* appeared to have seasonal adaptations in body mass, food intake, body fat mass, thermogenic capacity, biochemical processes, gene expression levels, and hormonal concentrations. This indicated that the maximum physiological capacity appeared under the most extreme conditions (winter-like or low temperature/short photoperiod conditions) in *T. belangeri*. In particularly, body mass, serum leptin and biochemical processes were involved in regulating the physiological capacity (thermoregulation) during the winter-like conditions. The maximum physiological capacity plays a role to reply in winter-like condition of *T. belangeri*.

### Seasonal variations in body fat mass, thermogenic capacity and regulating appetite

In previous research, the body mass, RMR, and NST increased under winter conditions in tree shrews^46^. In this study, there was significant seasonal variation in the body fat mass in tree shrews, which was highest in winter and lowest in summer. This differed from other species, such as *Microtus maximowiczii*^53^, *Microtus pennsylvanicus*^54^, *Microtus ochrogaster*^55^, *A. chevrieri*^56^ and *Apodemus draco*^57^, which showed that body fat mass decreased when exposed to winter-like conditions. Small mammals increase thermogenic capacity to cope with cold stress^24^,^36^. There was significant seasonal variation in thermogenic capacity, as the M_t_P content, COX activity, and ST_4_ mitochondrial respiration all increased. UCP1 content was higher in winter than in summer, which was a consistent with previous studies of *Ochotona curzoniae*^58^, *Acomys russatus*^59^, *A. chevrieri*^56^ and *A. draco*^57^. The NST level was positively associated with the UCP1 content. The results showed that T_4_ 5′-D II activity was significantly higher in winter than in summer, which was responsible for the production of T_4_ in the thyroid gland and its transformation into T_3_ in the peripheral tissues. T_3_ concentration was significantly higher in winter than in summer, and there was a similar seasonal change trend for T_3_, NST, and UCP1. T_4_, the primary thyroid-product, is a relatively inactive status until transformed into T_3_ by deiodination^60^, and TSH stimulates the concentrations circulating between T_3_ and T_4_^61^. Thyroid hormones are a major modulator of cold-induced NST^62^: T_3_, a unique active form of thyroxine, positively influences the expression of the UCP1 gene^27^. The concentration of testosterone and testicular mass was significantly higher in summer and autumn (the breeding seasons is range from March to June)^63^ than in winter, which is associated with protecting the territory and competing for mates^64^. However, these results indicated that variations in thermogenesis was not only regulated by endocrine regulation, but also depended on the changes in physiological requirements during the breeding season.

Leptin plays a role in energy balance with fluctuations in climate conditions in *T. belangeri*^46^. There was a significant decreased in serum leptin during winter in tree shrews, however, the food intake and body fat mass increased in the same period. During experimental administration of exogenous leptin, field voles appeared to have higher sensitivity to the short photoperiod than the long photoperiod, which indicated increasing leptin sensitivity may play a key role in their ability to survive winter-like conditions^15^. Food intake and energy expenditure are associated with the balance between POMC/CART and NPY/AgRp neuronal activity^39^. Under food deprivation conditions, the mRNA levels of both NPY and AgRp increased, however, POMC mRNA levels decreased^65^,^66^. Injection of leptin into the abdomen regulated the hypothalamic POMC mRNA^67^, with a consequent decrease in expression of POMC mRNA during fasting, or loss of the leptin signal^68^. In this study, food intake and NPY mRNA levels were higher in the winter than in the summer, although POMC and CART mRNA levels were lower in winter than in summer. The POMC and CART mRNA expression levels were similar to the serum leptin levels, but opposite to food intake, which indicated that serum leptin decreased under winter conditions in tree shrews, reducing the inhibition of appetite by POMC and CART. The low serum leptin levels also increased expression of NPY mRNA, increasing appetite and food intake in tree shrews. As a signal, leptin controls food intake by regulating the balance between POMC and CART and NPY and AgRp, it was similar as the previous researches, but there were different strategies accompanied by a decrease in serum leptin levels, (1). NPY/AgRp mRNA expression were significantly increased and POMC/CART was significantly decreased in *E. olitor*^23^, (2). AgRP mRNA expression was significantly increased in *L. brandtii*^69^, (3). NPY mRNA expression was significantly increased in *A. chevrieri*^40^, and (4). on changes in all gene mRNA expression in *Cricetulus barabensis*^70^.

### Roles of photoperiod and/or temperature in thermogenesis capacity

Both short photoperiod and low temperature were important environmental factors in a previous report, and influenced the increase in body mass, food intake, thermogenic capacity and digested energy in tree shrews^46^. The results of this study implicated a key role of the adipostatic signal in regulating body mass and serum leptin levels during short photoperiods or cold in *T. belangeri* (Table 3), as decreased serum leptin levels during both short photoperiods and cold were observed in acclimated in tree shrews. Serum leptin decreased during short photoperiod acclimation, and increased during the long photoperiod course in *A. draco*^71^. There are different responses to short photoperiod, *E. miletus* and *A. chevrieri* appear a lower serum leptin and body mass over 28 days of acclimation during short photoperiods than in long photoperiods^72^,^73^. However, the increase in serum leptin was associated with increasing body mass under short photoperiod acclimated *D. groenlandicus*^74^. Leptin is a signal of starvation and increases food intake in rats which living in winter-like conditions, such as short photoperiods and low temperatures^75^. In the present study, there was a significant increases in food intake after 28 days during short photoperiod/cold acclimation, which was similar to previous reports of short-photoperiod- or cold-acclimated rats and other small mammals, such as *Rattus norvegicus*^76^,^77^, *P. sungorus*^78^, *L. brandtii*^21^, *M. unguiculatus*^21^ and *M. maximowiczii*^53^. During the breeding season, there were high serum leptin levels, with an increase in food intake in *P. sungorus*^6^. In fact, leptin sensitivity increases during short-photoperiod conditions, as it mediates food intake via the hypothalamic suppression of cytokine signaling^79^. These results suggested that tree shrews were more sensitive to temperature than to photoperiod during extreme conditions (Table 3). However, over the seasonal cycle, the photoperiod is a more reliable environmental cue than temperature, as *P. sungorus*^80^, *Leuresthes tenuis*^81^ and plants^82^. This may be due to this species originating from tropical climates where the photoperiod variation is small, the species keep the ancestral physiological traits within a tropical and subtropical range.

The variation in individual thermogenesis was associated with biochemical markers, including mitochondrial protein content, COX activity, and UCP1 content. After 28 days acclimation, there was an increase in UCP1 (44.86%) content during the short photoperiod and cold group (Table 3). The increase in NST could be attributed to the increased expression of UCP1, as T_3_ can stimulate the transcription of UCP1^83^, and T_3_ and T_4_ concentration is associated with T_4_ 5′-DII activity. We found there were differences in serum T_3_ and T_4_ content in *T. belangeri*, as the serum T_3_ was higher in the short photoperiod and cold group than in the long photoperiod and warm group, although the serum T_4_ was just contrary to T_3_, which was lower in cold conditions than in warm. The TSH increased under short photoperiod and cold acclimation (Table 3). T_3_ and T_4_ concentrations were associated with T_4_ 5′-DII activity, and the T_4_ 5′-DII activity was higher in the short photoperiod in cold group than in other groups. The high T_4_5′ DII activity elevated the rate of conversion from T_4_ to T_3_, and the TSH level gained, resulting in decreased T_4_ level. The high TSH level increased release of T_4_ from the pituitary gland. This process maintains the high T_3_ level, which keeps the high expression levels of UCP1 and NST or thermogenesis to cope with the winter-like conditions. The results indicated a consequence of higher sensitivity to cold in tree shrews. There is a controversial relationship between leptin and UCP1. Exogenous leptin induces thermogenesis by elevating the expression of UCP1 mRNA^84^,^85^. However, BAT thermogenic capacity was reduced (in other words, the UCP1 decreased) resulting in the administration of leptin to cold-acclimated rats^71^, which is similar to the short photoperiod condition in this study. After 28 days of acclimation, serum leptin decreased but BAT thermognic capacity increased in tree shrews during cold exposure. This indicated that hormone, including leptin and thyrotropin, can regulate BAT thermogenesis by regulating the UCP 1 content.

In conclusion, environmental factors play key roles in the regulation of seasonal adaptation of body mass, thermogenesis, and food intake in wild small mammals. Our results supported the hypothesis that the extreme environmental conditions induce the physiological capacity by thermogenesis, hormone, gene expression, and biochemical processes. Decreasing serum leptin levels were associated with food intake, and thermogenesis increased to cope with winter-like conditions or the winter season (Tables 2 and 3). Winter-like conditions (Cold and/or short photoperiod condition) decreased the serum leptin level, but thermogenesis capacity increased to elevated physiological capacity, and resulted in increased gene expression of NPY mRNA, but POMC and CART mRNA expression decreased (Supplementary Figure S1). As a consequence of increased appetite and food intake, body mass was elevated and thermogenesis increased in tree shrews. The patters are different with the Palaearctic species, as *M. unguiculatus*^86^, the hibernating species, as *D. gliroides*^28^, and the *Sminthopsis crassicaudata*^87^,^88^. This was associated with changes in other traits, including biochemical processes and hormones. Seasonal variation and acclimation, showed that temperature and photoperiod play a key role in the regulation of thermogenesis capacity in *T. belangeri*.

## Additional Information

**How to cite this article:** Zhang, L. *et al*. Role of thermal physiology and bioenergetics on adaptation in tree shrew (*Tupaia belangeri*): the experiment test. *Sci. Rep.* **7**, 41352; doi: 10.1038/srep41352 (2017).

**Publisher's note:** Springer Nature remains neutral with regard to jurisdictional claims in published maps and institutional affiliations.

## Supplementary Material

Supplementary Information

## Figures and Tables

**Table 1:** Gene-Specific Primers Used for Real-Time RT-PCR.

Primer	Oligonuncleotide sequence (5′ to 3′)	Production size (bp)
NPY (forward)	TCGCTCTGTCCCTGCTCGTGTG	133
NPY (reverse)	TCTCTTGCCGTATCTCTGCCTGGTG	
POMC (forward)	CCTGTGAAGGTGTACCCAATGTC	276
POMC (reverse)	CACGTTCTTGATGATGGCGTTC	
CART (forward)	AGAAGAAGTACGGCCAAGTCC	50
CART (reverse)	CACACAGCTTCCCGATCC	
Bata-actin (forward)	ATGGTCAGGTGATCACCATTGGCAA	170
Bata-actin (reverse)	TTCTGCATTCTGTCAGCAAT	

NPY, neuropeptide Y; POMC, pro-opio-melanocortin; CART; cocaine- and amphetamine-regulated transcript.

**Table 2:** Thermogenic properties in liver, brown adipose tissue and hormones in tree shrews under different seasons.

	Spring (n = 8)	Summer (n = 10)	Autumn (n = 9)	Winter (n = 8)	*F*
Body mass	117.6 ± 5.8^b^	105.4 ± 4.8^c^	112.7 ± 2.6^b^	125.69 ± 4.3^a^	3.46^*^
***Brown adipose tissue***					
Mass (g)	0.57 ± 0.07^b^	0.43 ± 0.07^c^	0.52 ± 0.06^b^	0.63 ± 0.05^a^	2.98^*^
% body mass	0.48 ± 0.03	0.41 ± 0.02	0.46 ± 0.07	0.50 ± 0.06	0.96^*ns*^
TP (mg·g^−1^)	26.41 ± 1.20^a^	17.39 ± 0.97^c^	21.36 ± 1.02^b^	28.44 ± 0.81^a^	4.21^**^
M_t_P (mg·g^−1^)	10.21 ± 0.94^ab^	6.92 ± 1.02c	9.58 ± 0.84^b^	12.98 ± 0.95^a^	2.98^*^
ST_4_ (nmol O_2_ mg^−1^ MtP min^−1^)	9.89 ± 0.45	8.62 ± 0.15	9.11 ± 0.36	10.95 ± 0.26	3.06^*^
UCP 1 (RU/total BAT tissue)	1.29 ± 0.45^b^	1.00 ± 0.21^c^	1.36 ± 0.19^b^	1.68 ± 0.36^a^	3.11^*^
COX (μg atoms Omin^−1^ mg^−1^ MtP)	925.6 ± 123.1^b^	468.5 ± 89.1^d^	795.6 ± 89.3^c^	1216.4 ± 112.3^a^	4.69^**^
T_4_ 5′-D II (pmol O_2_ ·mg^−1^ ·MtP min^−1^)	33.48 ± 3.5^b^	27.60 ± 2.9^c^	31.98 ± 1.9^b^	39.48 ± 3.1^a^	3.85^*^
***Liver***					
Mass (g)	5.32 ± 0.36^ab^	4.67 ± 0.48^bc^	5.03 ± 0.24^b^	5.78 ± 0.56^a^	3.42^*^
% body mass	4.45 ± 0.27	4.43 ± 0.26	4.46 ± 0.31	4.60 ± 0.15	2.46^*ns*^
TP (mg·g^−1^)	91.25 ± 0.47^b^	82.81 ± 3.26^d^	86.59 ± 0.64^c^	97.52 ± 4.23^a^	3.48^*^
M_t_P (mg·g^−1^)	33.61 ± 0.21^b^	28.41 ± 1.95^c^	34.11 ± 0.22^b^	38.33 ± 2.11^a^	3.01^*^
ST_4_ (nmol O_2_ mg^−1^ MtP min^−1^)	27.27 ± 0.96^b^	24.57 ± 1.01^c^	25.98 ± 0.84^c^	29.25 ± 1.48^a^	3.74^*^
COX (μg atoms O min^−1^ mg^−1^ MtP)	79.24 ± 3.12^b^	76.64 ± 4.31^c^	80.14 ± 2.11^b^	85.02 ± 2.36^a^	3.65^*^
***Hormones***					
Tri-iodothyronine (T_3_, ng·ml^−1^)	1.13 ± 0.13^ab^	0.74 ± 0.08^c^	0.98 ± 0.11^b^	1.38 ± 0.19^a^	4.15^*^
Thyroxine (T_4_, ng·ml^−1^)	16.12 ± 0.68	16.70 ± 0.62	15.21 ± 0.56	14.79 ± 0. 61	1.96^*ns*^
T_3_/T_4_ (×100)	7.01 ± 0.22^b^	4.43 ± 0.30^d^	6.44 ± 0.19^c^	9.33 ± 0.28^a^	5.13^**^
Thyroid-stimulating hormone (TSH, ng·ml^−1^)	0.83 ± 0.09	0.85 ± 0.08	0.79 ± 0.15	0.75 ± 0.11	0.95^*ns*^

Different superscripts in each row means significantly different (Tukey’s post hoc test, α = 0.05; a > b > c) among seasons.

Statistical analyses: ^*^P < 0.05, ^**^P < 0.01, ^*ns*^*P* > 0.05.

**Table 3:** Influences of photoperiod (P) and/or temperature (T) thermogenic properties in liver, brown adipose tissue and hormones in tree shrews.

	Short photoperiod		Long photoperiod		The results of statistical analyses		
	5 °C (n = 10)	30 °C (n = 10)	5 °C (n = 10)	30 °C (n = 10)	*Temperature*	*Pohotoperiod*	*T* X *P*
Body mass	136.26 ± 3.86^a^	102.38 ± 2.68^c^	125.36 ± 1.95^b^	98.56 ± 2.56^c^	12.56^**^	2.26^*ns*^	0.06^*ns*^
***Brown adipose tissue***							
Mass (g)	0.73 ± 0.08^a^	0.36 ± 0.05^c^	0.54 ± 0.03^b^	0.29 ± 0.07^c^	3.12^*^	0.56^*ns*^	2.46^*ns*^
% body mass	0.54 ± 0.05^a^	0.35 ± 0.08^b^	0.43 ± 0.04^ab^	0.29 ± 0.03^c^	3.16^*^	3.48^*^	4.21^*^
TP (mg·g^−1^)	40.86 ± 1.6^b^	48.3 ± 2.1^a^	22.7 ± 2.6^c^	8.57 ± 0.9^d^	3.26^*^	3.77^*^	3.12^*^
M_t_P (mg·g^−1^)	17.12 ± 1.8^a^	8.65 ± 1.6^c^	12.1 ± 1.9^b^	4.33 ± 1.3^d^	5.63^*^	6.31^**^	6.12^**^
ST_4_ (nmol O_2_ mg^−1^ MtP min^−1^)	65.25 ± 2.3^a^	16.8 ± 1.8^c^	47.78 ± 3.4^b^	15.9 ± 2.1^c^	3.87^*^	4.11^*^	2.98^*^
UCP 1 (RU/total BAT tissue)	3.54 ± 0.47^a^	1.82 ± 0.53^b^	2.06 ± 0.82^b^	1.00 ± 0.39^c^	3.15^*^	5.69^**^	6.45^**^
COX (μg atoms O min^−1^ mg^−1^ MtP)	3726 ± 207^a^	586.7 ± 89.1^c^	2211 ± 109^b^	475.1 ± 75.3^c^	3.78^*^	3.28^*^	3.69^*^
T_4_ 5′-D II (pmol O_2_ ·mg^−1^ MtP min^−1^)	47.46 ± 2.4^a^	8.16 ± 1.28^c^	23.8 ± 2.4^b^	4.15 ± 1.9^d^	9.14^**^	8.21^**^	6.89^**^
***Liver***							
Mass (g)	7.94 ± 0.46^a^	4.98 ± 0.32^c^	5.67 ± 0.54^b^	4.35 ± 0.34^c^	3.76^*^	5.67^*^	3.54^*^
% body mass	5.83 ± 0.46^a^	4.86 ± 0.34^b^	4.52 ± 0.67^c^	4.41 ± 0.59^c^	3.13^*^	3.84^*^	4.26^*^
TP (mg·g^−1^)	122.6 ± 11.3^a^	80.6 ± 7.9^b^	92.3 ± 12.5^b^	59.5 ± 6.3^c^	3.98^*^	3.15^*^	3.94^*^
MtP (mg·g^−1^)	42.7 ± 5.3^a^	27.5 ± 6.4^b^	25.9 ± 3.5^a^	27.1 ± 4.1^b^	3.87^*^	4.21^*^	4.09^*^
ST_4_ (nmol O_2_ mg^−1^ MtP min^−1^)	41.89 ± 2.3^a^	23.5 ± 3.2^b^	24.7 ± 4.1^b^	18.7 ± 3.7^c^	4.97^*^	3.59^*^	4.11^*^
COX (μg atoms Omin^−1^·mg^−1^MtP)	152.6 ± 10.1^a^	56.8 ± 9.8^c^	98.5 ± 8.4^b^	36.9 ± 4.8^c^	4.36^*^	4.65^*^	3.79^*^
***Hormones***							
Tri-iodothyronine (T_3_, ng·ml^−1^)	1.93 ± 0.18^a^	0.69 ± 0.07^c^	1.22 ± 0.04^b^	0.71 ± 0.05^c^	4.68^*^	5.32^**^	3.97^*^
Thyroxine (T_4_, ng·ml^−1^)	30.53 ± 1.94^b^	44.47 ± 2.47^a^	31.41 ± 2.12^b^	46.24 ± 3.88^a^	4.16^*^	2.94^*ns*^	3.02^*ns*^
T_3_/T_4_ (×100)	6.32 ± 0.18^a^	1.55 ± 0.24^c^	3.88 ± 0.43^b^	1.54 ± 0.56^c^	4.36^*^	0.23^*^	0.15^*^
Thyroid-stimulating hormone (TSH, ng·ml^−1^)	1.18 ± 0.18^a^	0.76 ± 0.13^b^	0.81 ± 0.12^b^	0.49 ± 0.18^c^	5.08^*^	3.92^*^	1.23^*ns*^
Serum leptin (ng·ml^−1^)	4.94 ± 0.97^b^	10.64 ± 1.71^a^	5.46 ± 1.12^b^	10.87 ± 2.011^a^	4.45^*^	2.54^*ns*^	1.23^*ns*^

Results are presented as mean ± SME. Means with different letters differ significantly (Tukey’s post hoc test, α = 0.05; a > b > c) among treatment.

Statistical analyses: ^*^P < 0.05, ^**^P < 0.01, ^*ns*^*P* > 0.05.
