# Supplementary material for: Role of thermal physiology and bioenergetics on adaptation in tree shrew (Tupaia belangeri): the experiment test
Source: Sci Rep. 2017 Feb 1;7:41352. doi: 10.1038/srep41352 (PMC5286505; doi:10.1038/srep41352)
Supplement: Supplementary Information [file srep41352-s1.pdf]

Role of thermal physiology and bioenergetics on adaptation in tree shrew (*Tupaia belangeri*): the experiment test

Lin Zhang [†, §], Fang Yang [‡, §], Zheng-kun Wang [†, *], Wan-long Zhu [†, *]

[†] Key Laboratory of Ecological Adaptive Evolution and Conservation on Animals-Plants in Southwest Mountain Ecosystem of Yunnan Province Higher Institutes College, School of Life Sciences, Yunnan Normal University, Kunming 650500, China

[‡] School of Laboratory Medicine, Hubei University of Chinese Medicine, Wuhan 430065, China

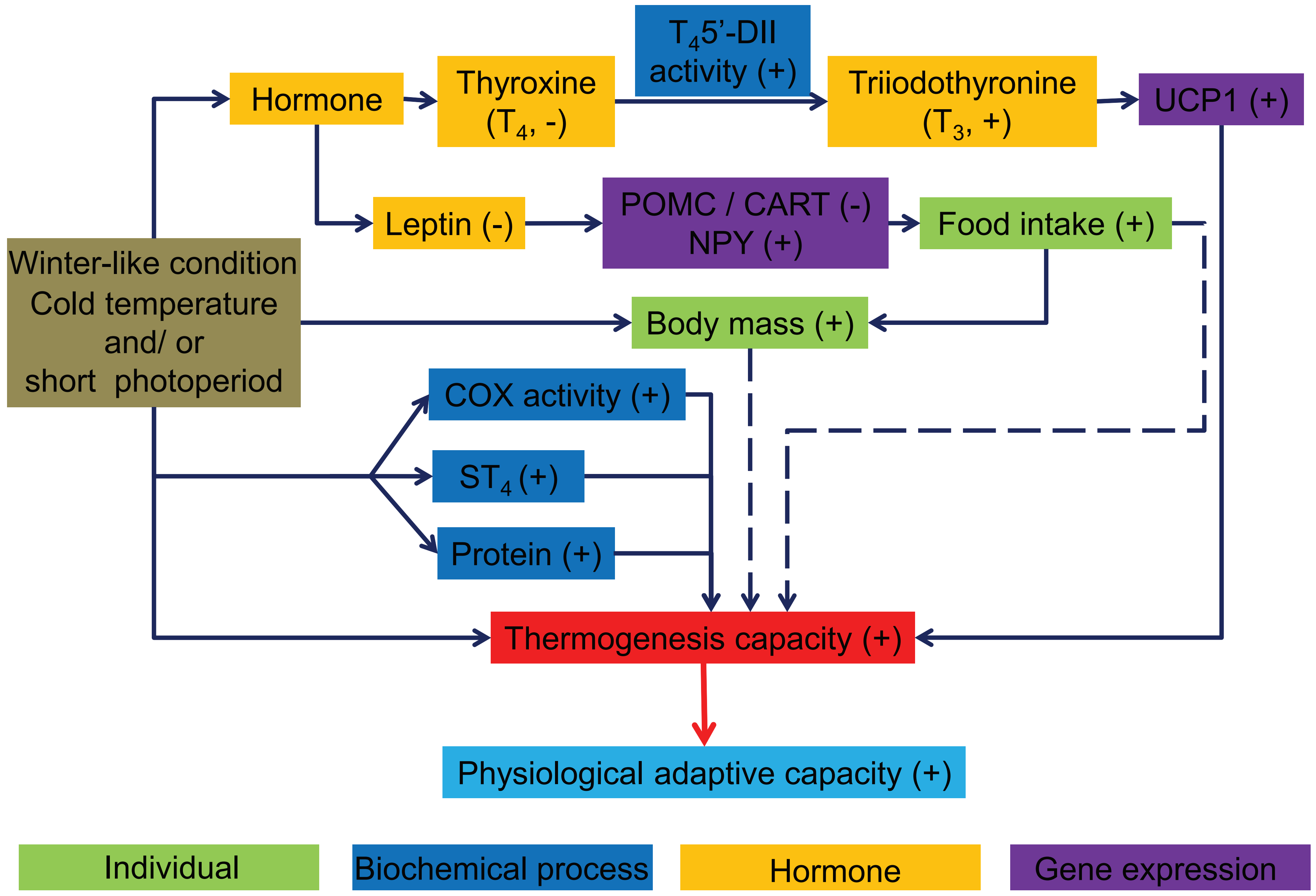
Hormone
Thyroxine (T4, -)
T4 5'-DII activity (+)
Triiodothyronine (T3, +)
UCP1 (+)
Leptin (-)
POMC / CART (-) NPY (+)
Food intake (+)
Winter-like condition Cold temperature and/ or short photoperiod
Body mass (+)
COX activity (+)
ST4 (+)
Protein (+)
Thermogenesis capacity (+)
Physiological adaptive capacity (+)
Individual
Biochemical process
Hormone
Gene expression
